# Whole-genome identification of *LdELF4s* and analysis of their expression response to diurnal temperature variations in *Lilium davidii* var. *willmottiae* (E. H. Wilson) raffill

**DOI:** 10.3389/fpls.2025.1683733

**Published:** 2026-01-07

**Authors:** Xiaohui Ma, Ying Li, Xudong Guo, Lingyu Meng, Yinquan Wang, Junji Su, Ling Jin

**Affiliations:** 1 Gansu University of Chinese Medicine, Lanzhou, Gansu, China; 2 Gansu Pharmaceutical Industry Innovation Research Institute, Gansu University of Chinese Medicine, Lanzhou, Gansu, China; 3 State Key Laboratory of Aridland Crop Science, College of Life Science and Technology, Gansu Agricultural University, Lanzhou, China

**Keywords:** *Lilium davidii var. willmottiae*, diurnal temperature variation, *ELF4* gene family, genome-wide identification, gene expression

## Abstract

**Introduction:**

Lanzhou lily (*Lilium davidii var. willmottiae*), a medicinal and edible plant endemic to China, synthesizes bioactive compounds regulated by diurnal temperature variations (DIFs). *ELF4* family genes, key regulators coordinating endogenous rhythms with external changes, are unexplored in this species.

**Methods:**

We analyzed nuclear–cytoplasmic phylogeny across 11 species and identified *ELF4* homologs. Phylogenetic, collinearity, cis-element, and expression pattern analyses were conducted for Lanzhou lily *ELF4s* (*LdELF4s*). qRT–PCR assessed *LdELF4* expression under different DIFs (20/20°C, 25/10°C, 20/5°C day/night), and corresponding phenotypes were evaluated.

**Results:**

Cytonuclear discordance was observed within *Lilium*. 62 *ELF4* homologs were identified and classified into four subfamilies. Six *LdELF4s* were located on chromosomes LG01, LG08, LG09, LG12, showing structural variation compared to *Lilium sargentiae*. *LdELF4.1, LdELF4.3*, and *LdELF4.6* were significantly upregulated at 25/10°C on day 14, while *LdELF4.4* and *LdELF4.5* were downregulated at 20/5°C. *LdELF4.6* showed highest sensitivity. Phenotypically, 25/10°C and 20/5°C treatments delayed flowering by 12.5 and 7.8 days, respectively. The 25/10°C treatment increased stem diameter but reduced flower bud number.

**Discussion/Conclusion:**

*LdELF4.6* is a core candidate gene regulating DIF response in Lanzhou lily, with 25/10°C potentially optimal for bulb development. This study provides genetic resources for breeding climate-adaptive lilies.

## 1 Introduction

Global climate warming has led to an increased frequency of extreme weather events, and temperature fluctuations are unavoidable environmental stressors during plant growth. These fluctuations not only impair plant developmental processes but also significantly compromise yield and quality (Ding et al., 2020). Compared with plants cultivated under optimal diurnal temperature variation (DIF), those exposed to suboptimal daytime and nighttime temperatures (i.e., elevated nocturnal heat or chilling stress) have disrupted physiological functions (Shi et al., 2017; Prasad et al., 2011; Peraudeau et al., 2015; Stavang et al., 2005; Abbas et al., 2023). Deviations from the species-specific thermal optimum alter phenotypic plasticity and ecological adaptability through perturbations in endogenous hormone homeostasis, photosynthetic carbon partitioning, and carbon-nitrogen metabolic pathways (Stavang et al., 2005). This thermoresponsive phenomenon has been validated across multiple taxa. In tomato (*Solanum lycopersicum*), Chen et al (Chen et al., 2014; Huang et al., 2021; Mao et al., 2012). demonstrated that DIF modulation significantly enhances the fruit setting rate, individual fruit weight, and yield metrics. Intriguingly, cultivar-specific DIF thresholds were identified: the wild accession LA1781 presented maximum fitness at 8–10°C DIF during preanthesis, whereas the cultivated varieties LA2397 and LA0490 presented optimal performance at 6°C DIF. Postanthesis, the wild-type genotypes presented superior yield and fruit quality under 10°C DIF, in contrast with their cultivated counterparts, whose yield and fruit quality peaked at 8°C DIF (Li et al., 2015). Notably, a 9°C DIF regimen effectively mitigated high daytime temperature stress (35°C) by alleviating the suppression of hypocotyl thickening in seedlings (Huang et al., 2021). Similarly, synergistic interactions between DIF and vernalizing temperatures accelerated bolting and flowering in radish (*Raphanus sativus*), directly influencing reproductive transitions (Pei et al., 2018). Maize (*Zea mays*) exhibited heightened sensitivity to narrow DIF ranges: a 2°C reduction in DIF increased nocturnal respiratory losses by 37%, accompanied by 28% and 31% declines in nonreducing sugars and starch reserves, respectively, resulting in a 24% reduction in biomass (Sunoj et al., 2016). In addition to those of staple crop species, understanding thermoadaptation mechanisms in medicinal species holds substantial agroeconomic importance. While the impact of DIF on staple crops is well documented, its effects on high-value medicinal plants, which often derive their economic importance from specialized metabolites, remain less explored. Understanding thermoadaptation mechanisms in these species holds substantial agroeconomic importance. A prime example is the dual-purpose (medicinal-culinary) Lanzhou lily (*Lilium davidii* var. *willmottiae)*, a sweet cultivar variant of *L. davidii* (Xu et al., 2009). As the sole nonbitter member among China’s four major lily cultivars, it produces fleshy bulbs renowned for their gastronomic texture and pharmacological efficacy in nourishing pulmonary yin and fortifying spleen-stomach function (Liang, 2011). Field observations across its primary cultivation zones (Qilihe District of Lanzhou city and Jingyuan County of Baiyin city, Gansu Province) revealed that elevated DIF positively correlates with the biosynthesis of bioactive compounds, particularly soluble sugars, total phenolics, and flavonoids, in bulbs. Intriguingly, bulb sucrose content is significantly negatively correlated with annual maximum temperatures (Lu and Lin, 2023). Therefore, identifying the key genes that control lily perception of diurnal temperature differences is highly important for the adaptive growth of lilies in different environments. The observed physiological correlations between DIF and bulb quality in Lanzhou lily underscore the necessity of deciphering the underlying molecular mechanisms. Plants perceive and respond to DIFs through conserved genetic networks, among which ELF4 family genes play pivotal roles.

*ELF4*s (*early flowering 4* genes) are crucial for plants to coordinate endogenous rhythms with external environmental changes and serve as key regulatory factors connecting environmental signals to growth and development (Zhao et al., 2021). Among them, *ELF3* was identified as a core circadian clock component and a pivotal integrator of environmental signals. It was shown not only to perceive light signals but also to function as an important thermosensor, playing a significant role in plant responses to temperature changes (Zhu and Wang, 2024; Mizuno et al., 2014b; Kolmos et al., 2011). At dusk, *ELF3* acts as a scaffold protein, forming an evening complex (EC) with *ELF4* and *LUX* (*LUX ARRHYTHMO/PHYTOCLOCK 1*). The activity of the EC peaks at night, indicating a potent transcriptional repressor function (Zhao et al., 2021; Silva et al., 2020; Huang and Nusinow, 2016; Nieto et al., 2015; Nusinow et al., 2011). Both light and temperature were found to regulate the activity of the EC and its binding affinity to target genes. Moderately high temperatures inhibited EC activity, whereas moderately low temperatures increased it. This regulatory mechanism enables plants to perceive diurnal temperature fluctuations and precisely modulate downstream genes, thereby regulating plant growth and development (Mizuno et al., 2014a; Silva et al., 2020; Mizuno et al., 2014b). Concurrently, the *ELF4* family strongly regulated flowering. Previous studies have demonstrated that temperature signals indirectly regulate the expression of flowering pathway genes by influencing EC activity, consequently affecting flowering time (Mizuno et al., 2014b). Furthermore, the functions of the *ELF4* family are highly conserved and diverse. Despite considerable sequence divergence (<37% similarity), *ELF3* orthologs from monocots (such as *Brachypodium distachyon* and *Setaria viridis*) completely complemented the circadian and flowering phenotypes of *Arabidopsiself3* mutants and were able to form analogous ECs, indicating highly conserved molecular and physiological functions across angiosperms (Huang et al., 2017). In summary, *ELF4*s constitute core molecular modules through which plants perceive and integrate environmental signals. They precisely coordinate plant developmental rhythms to adapt to changing environments by regulating core gene expression, integrating light and temperature signals, and directly controlling key downstream genes involved in growth and development.

In this study, the phylogenetic relationships of *L. davidii* var. *willmottiae* and the expansion and contraction of orthologous groups were analyzed on the basis of nuclear and chloroplast genomes from different species. Interspecific evolutionary analysis and promoter cis-acting element analysis were performed specifically for the *LdELF4* family members. The tissue-specific expression patterns were subsequently analyzed on the basis of the transcriptome data, and six key candidate genes whose expression significantly differed under different day/night temperature conditions were identified via qRT–PCR. Furthermore, the influence of temperature treatment on the growth and development of Lanzhou lilies was clarified. These findings improve our understanding of *LdELF4*s and provide insights into novel genetic resources for the molecular breeding of lily varieties adaptable to climate change.

## 2 Materials and methods

### 2.1 Nuclear genome phylogenetic analysis and orthogroup evolutionary investigation

Twelve species (*Arabidopsis thaliana*, *Zea mays*, *Triticum aestivum*, *Solanum lycopersicum*, *Glycine max*, *Vitis vinifera*, *Populus trichocarpa*, *Physcomitrium patens*, *Allium sativum*, *Lilium davidii* var. *willmottiae*, *Lilium lancifolium*, and *Lilium sargentiae*) were selected as study subjects. Genome annotation files for these species were obtained from the phytozome database (https://phytozome-next.jgi.doe.gov/) and published literature. The coding sequences (CDSs) and protein sequences corresponding to the longest transcript isoforms were extracted using the FASTA Get Representative module in TBtools. Whole-genome orthologous group analysis was performed on the protein sequences of the 12 species using OrthoFinder2, and single-copy orthogroups (OGs) were screened for subsequent analyses. Multiple sequence alignment was conducted with MAFFT, followed by the trimming of conserved sites using trimAI. Single-copy gene sequences from each species were concatenated and integrated. A maximum likelihood (ML) phylogenetic tree was constructed using IQ-TREE, with *Physcomitrium patens* designated as the outgroup. The optimal substitution model was automatically selected using the “-m MFP” parameter. Node support values were assessed via ultrafast bootstrap (“-bb 1000”) and NNI optimization (“-bnni”).

Fossil calibration times for key nodes were retrieved from the TimeTree database (http://www.timetree.org/) and integrated into the nuclear genome-based phylogenetic tree. Site substitution rates were estimated using the codeml module in the PAML software package, and Bayesian molecular clock analysis was performed with the MCMCTree module. Two independent runs were conducted to verify the consistency of the results. Gene family expansion and contraction analyses were implemented in CAFÉ on the basis of the OG gene count matrix generated by OrthoFinder2 and the divergence time-calibrated phylogenetic tree. Models with K = 2–5 were compared, and the optimal evolutionary model was selected using the maximum likelihood value (-lnL) as the criterion.

### 2.2 Chloroplast genome phylogenetic analysis

The complete chloroplast genome sequences of the 12 species were retrieved from the NCBI Organelle Genome Database (https://www.ncbi.nlm.nih.gov/genome/organelle/). Multiple chloroplast genome alignment was performed using MAFFT, with conserved sites refined using trimAI. A ML phylogenetic tree was subsequently constructed with IQ-TREE (parameters consistent with those used in the nuclear genome analysis) to validate the evolutionary relationships of the plastid genome among the species.

### 2.3 Identification of *ELF4* family members across different species

The protein family Pfam ID PF07011.16 was determined by using the ELF4 protein sequence from *Arabidopsis thaliana* as a reference. On the basis of the hidden Markov model (HMM) sequence identifiers, a whole-genome search was conducted to identify all the *ELF4* family members. Redundant sequences were manually removed based on an E value threshold of less than 1e-10. The protein was verified as an *ELF4* family gene containing a DUF1313 domain through internal sequence analysis using the SMART database (https://smart.embl-heidelberg.de/). Additionally, the NCBI Conserved Domain Database (CDD) was used to validate the conservation of the *ELF4* family. The physicochemical properties of the ELF4 proteins were analyzed using the ProtParam tool available on the ExPASy website, and the subcellular localization of the *ELF4*s was predicted using the ProtComp tool provided by Softberry.

### 2.4 Phylogenetic tree construction of *ELF4* in different species

*ELF4* Pfam ID was used to identify *ELF4* family members in the genomes of Lanzhou lily (*L. davidii* var. *willmottiae*), *Lilium sargentiae* (*L. sargentiae*), *Gloriosa superba* (*G. superba*), *Arabidopsis* (*A. thaliana*), soybean (*G. max*), *Physcomitrium patens* (*P. trichocarpa*), tomato (*S. lycopersicum*), grape (*V. vinifera*), rice (*O. sativa*), maize (*Z. mays*) and garlic (*A. sativum*). The protein sequences were extracted using TBtools and aligned with ClustalW in MEGA11 software. A phylogenetic tree of the eleven species was constructed by the Jones–Taylor–Thornton (JTT) model, with the reliability of the tree evaluated by performing 1000 bootstrap replicates through the neighbor–joining (NJ) method.

### 2.5 Interspecific and intraspecific collinearity analysis

The complete-genome sequence and general feature format version 3 (gff3) annotation files of *Lilium davidii* var. *willmottiae* and *Lilium sargentiae* were downloaded from published literature, and the CDSs were extracted with TBtools. BEDtools was used to convert gff3 files into BED files, the seqkit tool was used to remove duplicate IDs from the BED files to obtain the whole-genome CDS set and BED file of each species, and the JCVI-compara subcommand in the jcvi toolset was used to generate anchor files. Finally, collinear plots were drawn using the jcvi.graphics.karyotype module.

### 2.6 Gene structure and cis-acting element analysis of *ELF4s*

MEME (https://meme-suite.org/meme/tools/meme) was used to forecast the conserved motif of *ELF4*s, the base number of the motifs was set to 6, the base width ranged from 6 to 50, and the MEME result was obtained by submitting files; the *ELF4s* conservative domain.txt file was obtained by using the CDD and visualized by integrating the above files and gff3 and Newick files using TBtools. The PlantCARE (https://bioinformatics.psb.ugent.be/webtools/plantcare/html/) website was used to predict cis-acting elements in the upstream 2000 bp promoter sequence of *ELF4*s, and the predicted results were subsequently analyzed and categorized statistically.

### 2.7 Analysis of transcriptome expression patterns in different tissues of Lanzhou lily

The reference genome and corresponding gene annotation files of Lanzhou lily were retrieved from prior studies. With the application of the gffread tool, we subsequently extracted the transcript sequences and simplified the sequence identifiers to create the final cDNA file. Afterward, we merged the cDNA with the genome sequences into a new file and employed Salmon to construct a mapping index. Next, we obtained the raw sequencing files of different Lanzhou lily tissues from public databases and used Salmon for transcript expression quantification. Finally, the tissue-specific expression profiles of the target genes were visualized in TBtools using transcripts per million (TPM) values.

### 2.8 Plant material processing and expression level analysis

Following the transplantation of Lanzhou lily seedlings into seedling trays, they were cultivated in a growth chamber set at a constant diurnal temperature of 20°C (20/20°C day/night) under a 12-h photoperiod (12 h light/12 h dark) until emergence. The uniformly developed seedlings were subsequently randomly assigned to three groups (n=10 per group): a control group maintained at 20/20°C day/night and two treatment groups exposed to diurnal temperature regimens of 25/10°C and 20/5°C. The treatment groups were subjected to their respective temperature regimens for 14 days. Plant tissue samples were collected at three time points: 7 days post-treatment initiation (7 dpt), 14 days post-treatment initiation (14 dpt), and 7 days post-treatment cessation (7 dptc). The samples were immediately flash-frozen in liquid nitrogen. The frozen samples were homogenized to a fine powder, and total RNA was extracted using a commercial kit. Following the assessment of RNA purity and concentration, the qualified RNA was reverse transcribed to complementary DNA (cDNA). The resulting cDNA was diluted to a working concentration of 50 ng/μl for use as a template in quantitative real-time PCR (qRT–PCR).

Gene-specific qPCR primers for six candidate genes (*LdELF4s*) and the reference gene (*LdActin*) were designed using Primer Premier 5 software (primer sequences are detailed in **Supplementary Table S1**). The stability of the reference genes across our experimental temperature conditions was validated prior to analysis. The transcript abundance of each candidate gene under the different diurnal temperature treatments was quantified using the QuantStudio™ 5 Real-Time PCR System. Relative gene expression levels were calculated from the raw cycle threshold (Ct) values using the comparative 2^^−ΔΔCt^ method, with normalization against the geometric mean of the reference genes. Significant differences in gene expression among the treatment groups at each time point were determined using one-way analysis of variance (ANOVA) followed by Duncan’s multiple-range test. The significance threshold was set at P < 0.05. The data are presented as the means ± standard deviations (SDs).

## 3 Results and analysis

### 3.1 Cytonuclear discordance during the evolution of lilies

To investigate the phylogenetic relationships among three lily species (*Lilium davidii* var. *willmottiae*, *Lilium sargentiae* and *Gloriosa superba*) during evolution, we aggregated 554,748 gene alignments from 12 species into 55,446 OGs, of which 5,603 OGs were shared across all 12 species. A nuclear genome-based phylogenetic tree constructed using 32 single-copy OGs revealed clear divergence between monocots and eudicots. Six monocot species (maize, rice, garlic and the three lilies) formed distinct lineages, with bulbous species clustering into a separate subclade. In contrast, the chloroplast genome phylogeny revealed highly conserved evolutionary relationships among bulbous species but exhibited significant topological discrepancies compared with those of other species. Compared with nuclear genomes, this nuclear-plastid phylogenetic approach may arise from maternal inheritance and distinct evolutionary rates/selection pressures governing chloroplast genomes. Both nuclear and plastid phylogenies consistently supported the close affinity between *Lilium davidii* var. *willmottiae* and *Gloriosa superba*.

**Figure 1:**
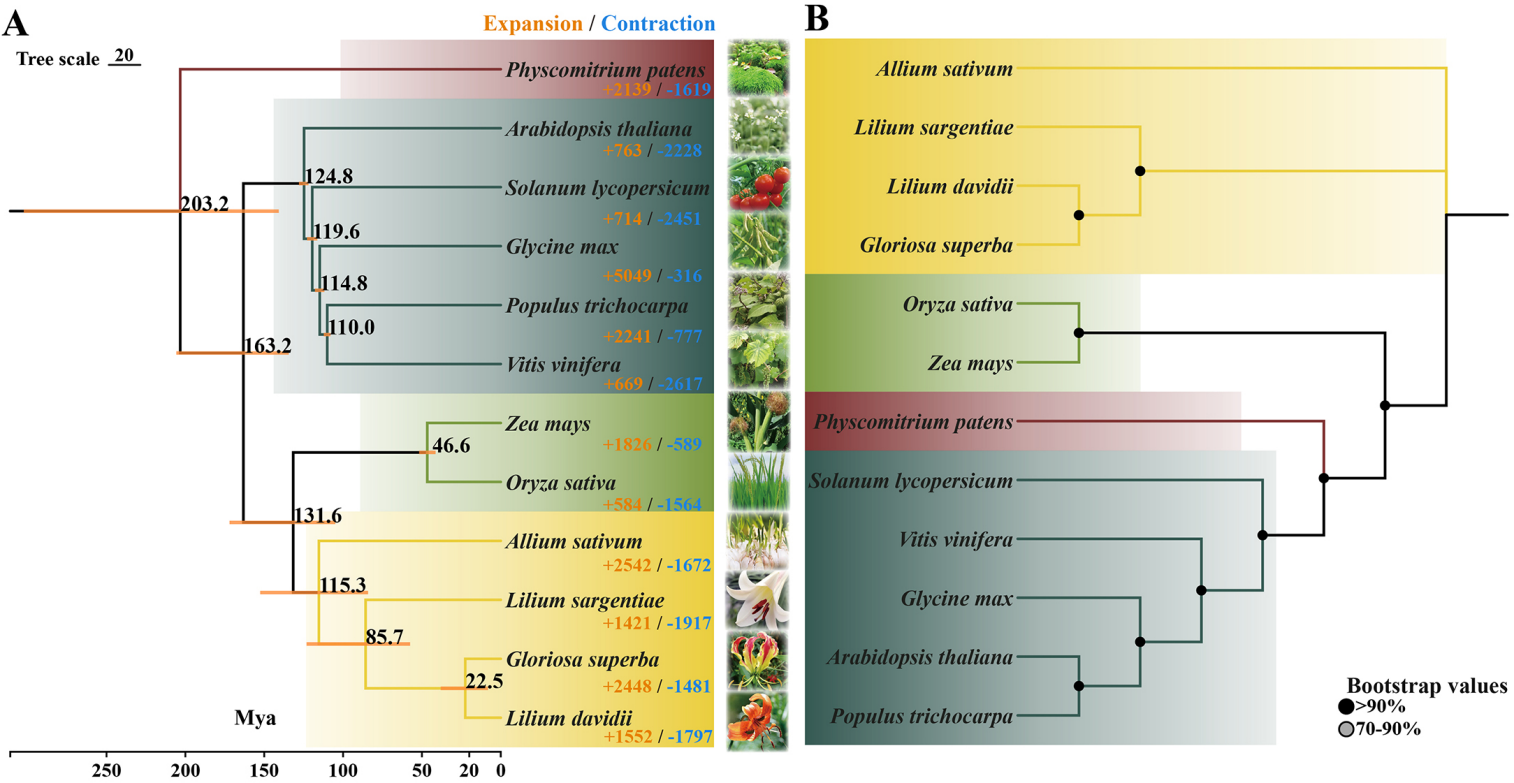
Phylogenetic relationships of 12 plant species (*Lilium* genus and other representative species) based on nuclear **(A)** and plastome **(B)** genome analysis.

Moreover, molecular clock modeling with fossil calibration was used to estimate the divergence timelines of *Lilium* species. The split between bulbous plants and Poaceae (grasses) occurred approximately 130 million years ago (Mya). The divergence of the *Lilium davidii* var. *willmottiae*/*Gloriosa superba* lineage from *Lilium sargentiae* was dated to 85.7 Mya, with subsequent species-level differentiation delayed until 22.5 Mya. Gene family dynamics analysis revealed convergent evolutionary patterns in *Lilium davidii* var. *willmottiae* (1,552 expanded and 1,797 contracted gene families) and *Lilium sargentiae* (1,421 expanded and 1,927 contracted families), whereas *Gloriosa superba* exhibited the opposite trend (**Figures 1A, B**). These disparities likely stem from heterogeneous distributions of whole-genome duplication (WGD) events, species-specific selection pressures, and potential biases introduced by variations in genome assembly completeness affecting gene family annotation accuracy.

### 3.2 Phylogenetic analysis of the ELF4 protein in lilies and other species

**Figure 2:**
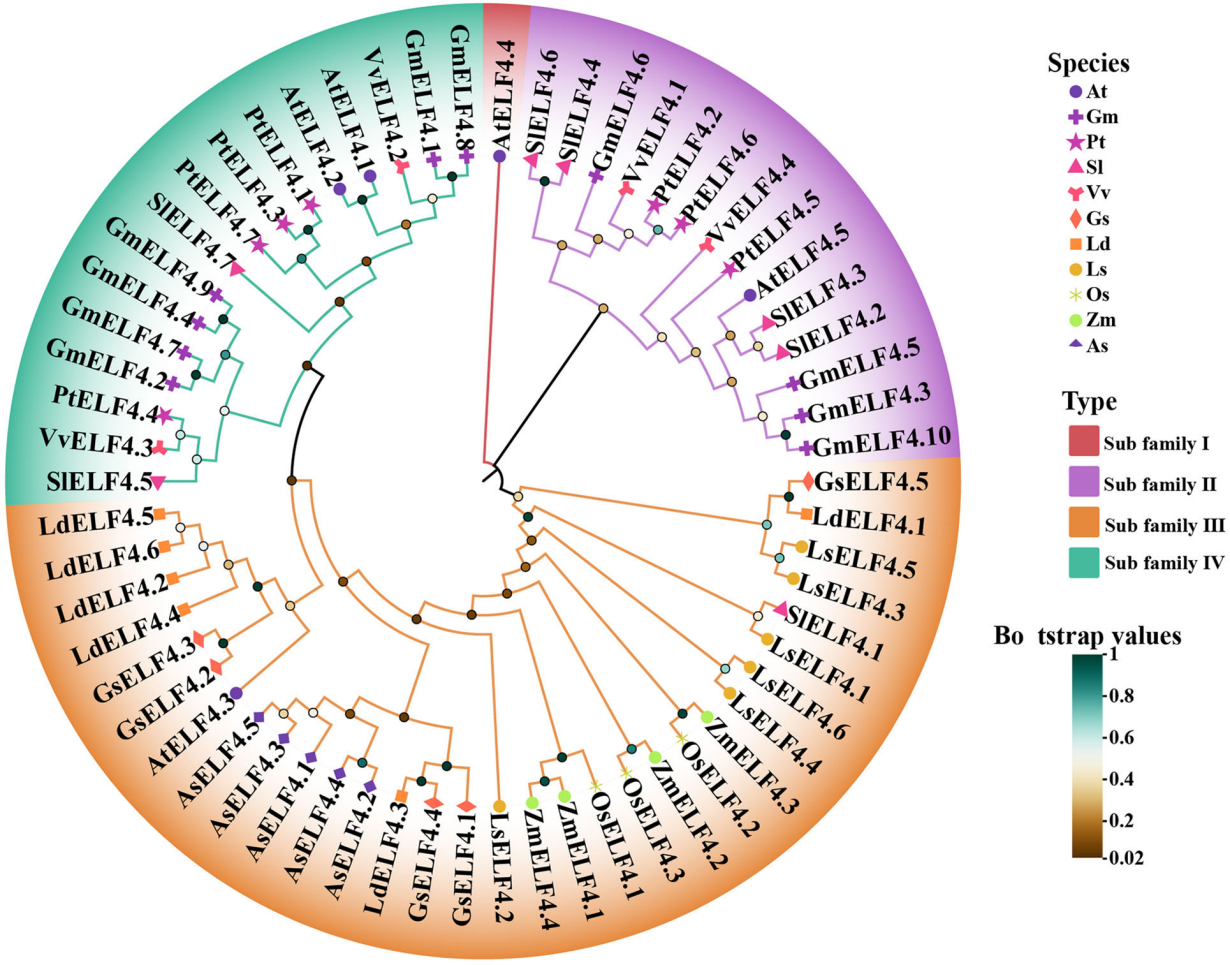
Phylogenetic analysis of the ELF4 protein among different species. Different symbols represent various species, different colors indicate distinct categories, and the self-expanding values are represented by a gradient from orange (low) to green (high).

To clarify the evolutionary process of lily genes in different species, the *ELF4* family was selected as the investigative target on the basis of gene expansion/contraction dynamics. We systematically identified 18 ELF4 homologs in lily by the Pfam ID (PF07011.16) HMM profile and the conserved structure (ELF4 domain). The gene IDs of 17 genes were renamed according to the genomic annotation file, including 6 in *L. davidii* var. *willmottiae* (*LdELF4.1*-*LdELF4.6)*, 6 in *L. sargentiae* (*LsELF4.1-LsELF4.6*), and 5 in *G. superba* (*GsELF4.1-GsELF4.5)*. In addition, we identified *ELF4* genes in eight other species, namely, *A. thaliana* (5), *G. max* (10), *P. trichocarpa* (7), *S. lycopersicum* (7), *V. vinifera* (4), *O. sativa* (3), *Z. mays* (4) and *A. sativum* (5). Through an analysis of the clustering relationships among these species, we clarified the relationships between the lilies and other species. The results of the evolutionary tree indicate that the 62 ELF4 proteins can be classified into four subfamilies. Subfamily III contains the greatest number of proteins, totaling 31 ELF4 proteins. These genes include 5 GsELF4s, 6 LdELF4s, 5 LsELF4s, and 5 AsELF4s from the genus *Lilium* as well as 4 ZmELF4s and 3 OsELF4s from monocotyledonous plants. Subfamilies II and IV contain similar numbers of proteins, primarily composed of ELF4s from dicotyledonous plants, such as GmELF4s, AtELF4s, VvELF4s and PtELF4s. In contrast, subfamily I contained the fewest proteins, with only one protein, AtELF4.4 (**Figure 2**). Our findings also demonstrate that *L. davidii* var. *willmottiae* is most closely related to *G. superba*, followed by *L. sargentiae*. Additionally, we observed that *L. davidii* var. *willmottiae* is relatively closely related to garlic, rice, and corn. These results suggest that the ELF4 proteins in lilies have remained relatively conserved during evolution and are more closely related to monocotyledonous ELF4 proteins than they are to those of dicotyledonous plants.

### 3.3 Chromosomal distribution and evolutionary analysis of *ELF4s* in lilies

**Figure 3:**
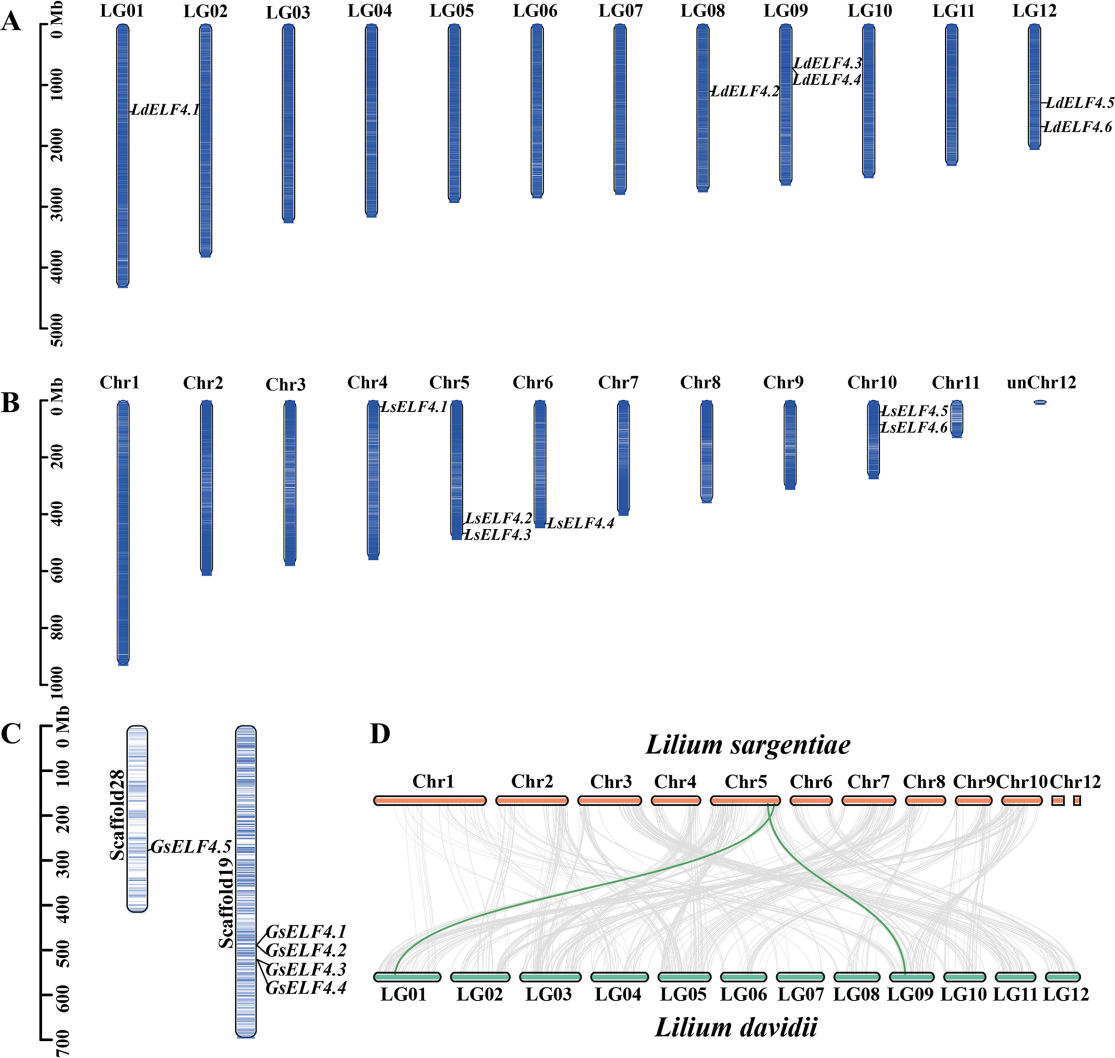
Evolutionary relationships of the *ELF4* gene family across Lilium species. **(A)** Chromosomal localization of *ELF4* family members in *L. davidii* var. *willmottiae*. **(B)** Chromosomal localization of *ELF4* family members in *L. sargentiae*. **(C)** Chromosomal localization of *ELF4* family members in *G .superba*. **(D)** Interspecific collinearity analysis of *ELF4* genes between *L. davidii* var. *willmottiae* and *L. sargentiae*.

To clarify the distribution of *ELF4s* in the genome, we visualized the chromosomal localization of the three lily species. The results showed that *LdELF4s* are primarily distributed on four chromosomes, all in the middle of the chromosomes. Among them, *LdELF4.1* and *LdELF4.2* were located on LG01 and LG08, respectively, whereas the remaining four genes were relatively concentrated on LG09 and LG12 (**Figure 3A**). *LsELF4s* are also distributed on four chromosomes and are located at both ends of the chromosomes, with a total of four genes present on chromosomes 5 and 10 (**Figure 3B**). *GsELF4s* were distributed on the large genomic fragments; *GsELF4.1 to GsELF4.4* were clustered on Scaffold 19, and *GsELF4.5* was located on Scaffold 28 (**Figure 3C**). To demonstrate the replication and rearrangement among lily species, we conducted a collinearity analysis on *L. davidii* var. *willmottiae* and *L. sargentiae.* These results indicate that chromosomal fragment translocation or fusion events may have occurred during the evolution of *L. sargentiae*; moreover, these events also serve as important markers of genomic differentiation (**Figure 3D**).

### 3.4 Analysis of the functional and structural characteristics of the *ELF4s* in lilies

To understand the overall characteristics of the lily *ELF4* family. The physicochemical properties of the gene family members of the three lily species were analyzed with ExPASy ProtParam (**Table 1**). The results revealed that the protein lengths in the various lily species significantly differed. The amino acid sequences from *G. superba* were the longest (100–890 aa), those from *L. sargentiae* were the shortest (69–198 aa), and those from *L. davidii* var. *willmottiae* were intermediate in length (100–139 aa). Isoelectric point analysis revealed that the isoelectric point distribution range of the *L. davidii* var. *willmottiae* proteins was relatively wide, with acidities ranging from 4.47 to 11.22 (3 acidic and 3 alkaline), whereas the proteins from *G. superba*, except for *GsELF4.5*, were weakly alkaline. All the proteins from *L. sargentiae* exhibited alkaline characteristics. The GRAVY index of all the members was negative, indicating that the proteins in this family are generally hydrophilic, among which the proteins from *L. sargentiae* were the most hydrophilic. The stability coefficients indicated that all the members of the ELF4 protein family in lilies are unstable except for *LdELF4.6* and *LdELF4.4*. Additionally, we found that none of the proteins in this family have transmembrane domains. Subcellular localization analysis and prediction indicated that the majority of ELF4 proteins were localized in the nucleus, *GsELF4.2* was localized in the chloroplast, and *LsELF4.3* and *LsELF4.4* were localized in the mitochondria and cytoplasm, respectively. These findings indicate that the ELF4 protein might play a key role in regulating gene expression. These findings provide insights into the structural characteristics and potential functional significance of ELF4 proteins in lily species.

**Table 1:** Analysis of the physicochemical properties of *ELF4*s.

Gene name	Gene ID	Number of amino acids	Theoretical pI	Instability index	Grand average of hydropathicity (GRAVY)	Subcellular localization
*LdELF4.1*	*Lily01G30500.1*	130	9.43	53.69	-0.765	nucleus
*LdELF4.2*	*Lily08G30610.1*	139	11.22	52.50	-0.846	nucleus
*LdELF4.3*	*Lily09G22210.1*	112	6.73	51.36	-0.702	nucleus
*LdELF4.4*	*Lily09G22220.1*	112	8.27	38.79	-0.451	nucleus
*LdELF4.5*	*Lily12G34110.1*	100	4.47	50.64	-0.317	nucleus
*LdELF4.6*	*Lily12G42870.1*	134	4.89	31.40	-0.515	nucleus
*GsELF4.1*	*lili00G207110.t1*	311	9.74	44.60	-0.786	nucleus
*GsELF4.2*	*lili00G207120.t1*	100	7.98	42.03	-0.182	chloroplast
*GsELF4.3*	*lili00G207730.t1*	176	8.80	53.08	-0.058	nucleus
*GsELF4.4*	*lili00G207740.t1*	890	9.45	41.20	-0.700	nucleus
*GsELF4.5*	*lili00G298160.t1*	115	4.87	47.66	-0.637	nucleus
*LsELF4.1*	*Gs04G014960*	86	8.37	51.10	-0.078	nucleus
*LsELF4.2*	*Gs05G140540*	116	7.93	51.56	-0.753	nucleus
*LsELF4.3*	*Gs05G157310*	119	12.14	90.31	-1.066	mitochondrion
*LsELF4.4*	*Gs06G091620*	69	9.98	74.77	-0.575	cytoplasm
*LsELF4.5*	*Gs10G027050*	198	9.68	73.15	-0.759	nucleus
*LsELF4.6*	*Gs10G056230*	110	7.93	48.49	-0.658	nucleus

### 3.5 Domain, gene structure and conserved motif analysis of the *ELF4s* in lilies

**Figure 4:**
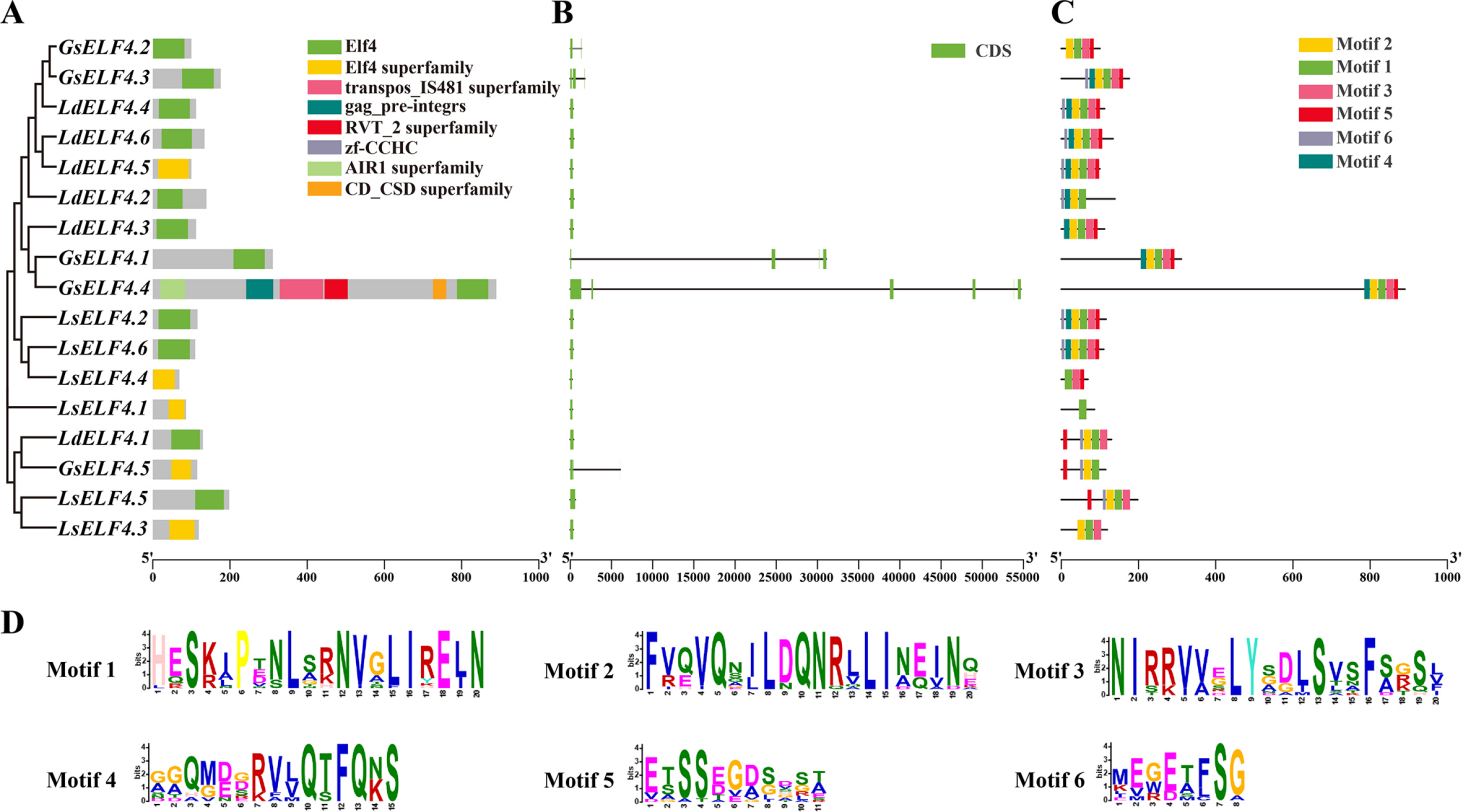
Analysis of lily gene structure, conserved domains and motifs. **(A)** Conserved domain analysis of ELF4 proteins in lily. **(B)** Gene structure analysis of *ELF4* genes in lily. **(C)** Conserved motif analysis of ELF4 proteins in lily. **(D)** Sequence logo representation of conserved motifs in lily ELF4 proteins.

Our in-depth analysis of the ELF4 domain, gene structure and conserved motif of lilies revealed these findings. Most *ELF4s* have a conserved ELF4 domain. In addition to *LdELF4.5*, which belongs to the ELF4 superfamily and may share some conserved domains, *GsELF4.4* also includes six other domains and may be involved in regulating the functions of other genes (**Figure 4A**). The genetic structure indicates that there is only one exon among the 12 *ELF4s*. Both *GsELF4.1* and *GsELF4.3* contain three exons, whereas *GsELF4.2* and *GsELF4.4* contain two and five exons, respectively (**Figure 4B**). These results indicate that members of the lily *ELF4* gene family have simple structures and encode proteins with single functions. Multiple sequence alignment was subsequently performed to identify conserved motifs, and the ELF4 domain structure was highly conserved. We found that the number of motifs in *ELF4s* ranged from 3 to 6 (**Figures 4C, D**). This observation highlights the key functional domains that are crucial to the biological functions of these genes. These analyses improve our understanding of the structural basis underlying the multiple functions of the *ELF4* family in lilies.

### 3.6 Analysis of cis-acting elements in the promoter region of *LdELF4*s in Lanzhou lily

**Figure 5:**
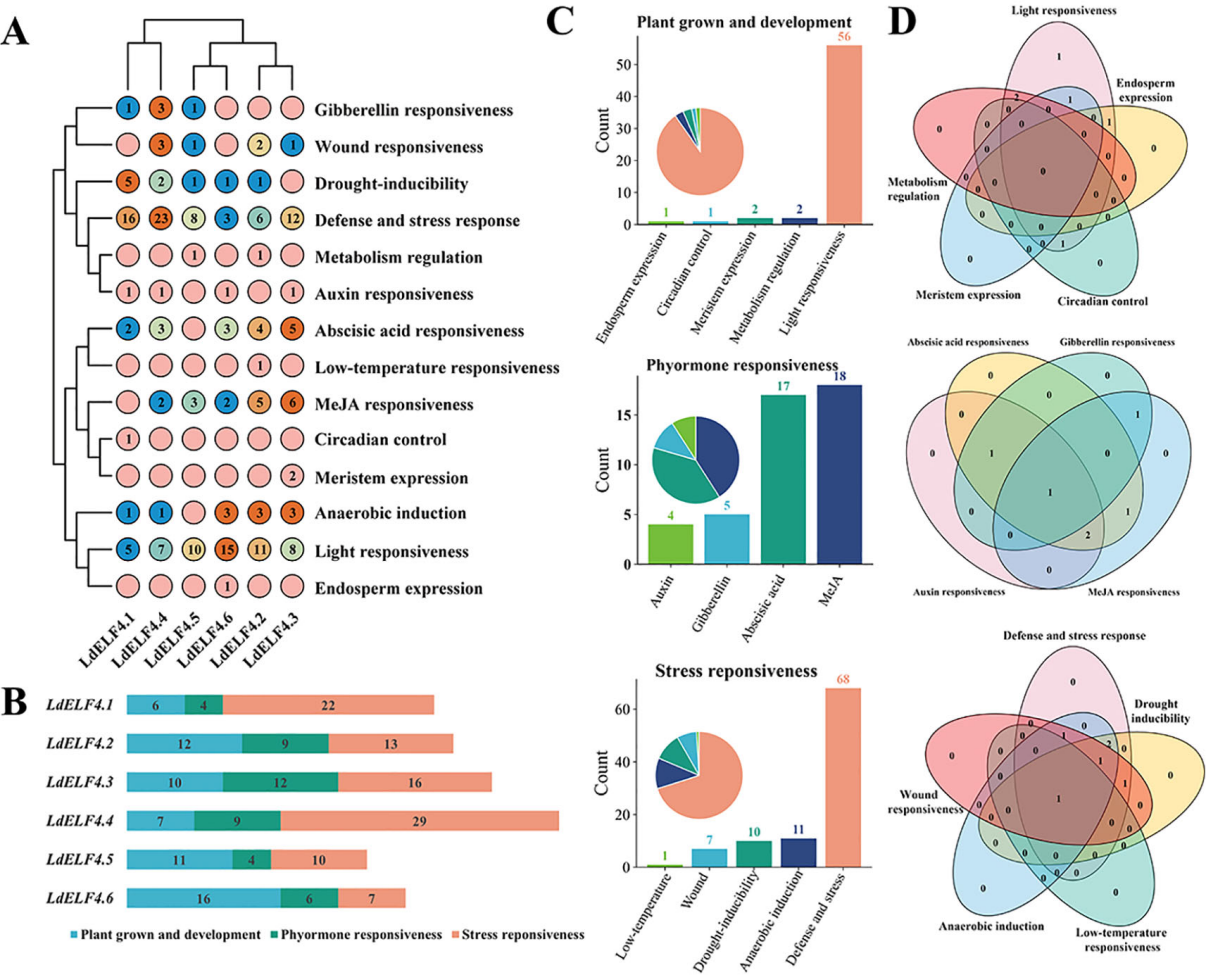
Analysis of the cis-acting elements in the Lanzhou lily promoter. **(A)** Heatmap of cis-regulatory element categories across *LdELF4*s. **(B)** Stacked bar plot of cis-regulatory element categories per *LdELF4*. **(C)** Quantification of cis-regulatory elements per functional category. **(D)** Venn diagram of *LdELF4*s overlapping by functional category.

To analyze the potential regulatory function of the cis-acting elements in the promoter region of *LdELF4*s in plant growth and development, in this study, six 2000 bp regions upstream of the transcription start sites (TSSs) of *LdELF4*s were extracted from the genome. The classification and statistics for the cis-acting elements revealed that these sequences can respond to 14 regulatory functions and are enriched primarily with three major types of functional elements—growth- and development-related elements (such as ARE, circadian and GCN4_motif); plant hormone response elements—abscisic acid (ABA) response elements, gibberellin (GA) response elements (including P-boxes and TATC-box), and methyl jasmonate (MeJA) response elements; and stress response elements (such as LTRs and TC-rich repeats). Notably, all the *LdELF4* promoters are equipped with light response elements and stress defense elements. Most promoters contain multiple plant hormone response modules; some sequences contain growth- and development-specific regulatory elements (such as circadian rhythm elements) (**Figures 5A, C**). The stress response elements account for the greatest proportion of elements in *LdELF4.1*, *LdELF4.2*, *LdELF4.3* and *LdELF4.4*, whereas in *LdELF4.5* and *LdELF4.6*, the elements with the greatest proportion are growth and development response elements (**Figure 5B**). In addition, we found that *LdELF4.4* is responsive to four hormonal stimuli, whereas *LdELF4.2* is responsive to five distinct abiotic stresses (**Figure 5D**).

In summary, the enrichment pattern of multiple elements in the promoter region suggests that *LdELF4* might participate in regulating the growth and development of plants through the integration of light signals, hormone pathways and stress response networks.

### 3.7 Analysis of Lanzhou lily phenotypes under various diurnal temperature differences

**Figure 6:**
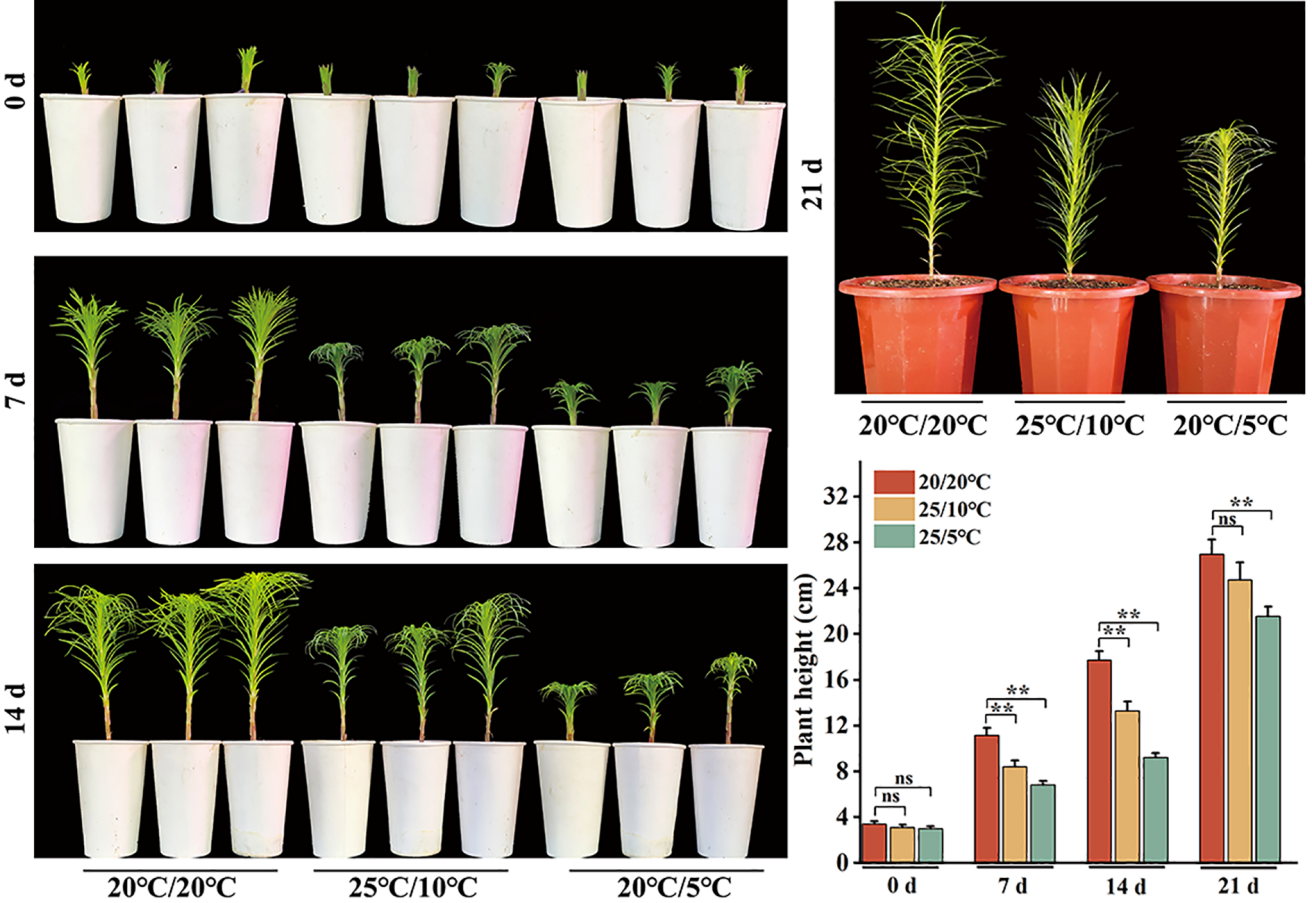
Analysis of lily plant height phenotypes under various diurnal temperature differences. Asterisks represent Student’s t tests for significant differences in statistical analysis: *P < 0.05; **P < 0.01; ns represents no significant difference.

Taking 20/20°C (day/night) as the standard germination temperature and setting it as the control, we observed that the plant height significantly increased compared with that in the two treatment groups at 25/10°C and 20/5°C. Under the 25/10°C treatment, the plants exhibited steady growth with a compact leaf morphology. In contrast, under the 20/5°C treatment, growth and development were delayed, resulting in a significant reduction in plant height. Additionally, seven days after the treatment was concluded, the difference in plant height between the treatment groups and the control group gradually diminished (**Figure 6**). These findings suggest that DIFs might influence plant growth and development.

**Figure 7:**
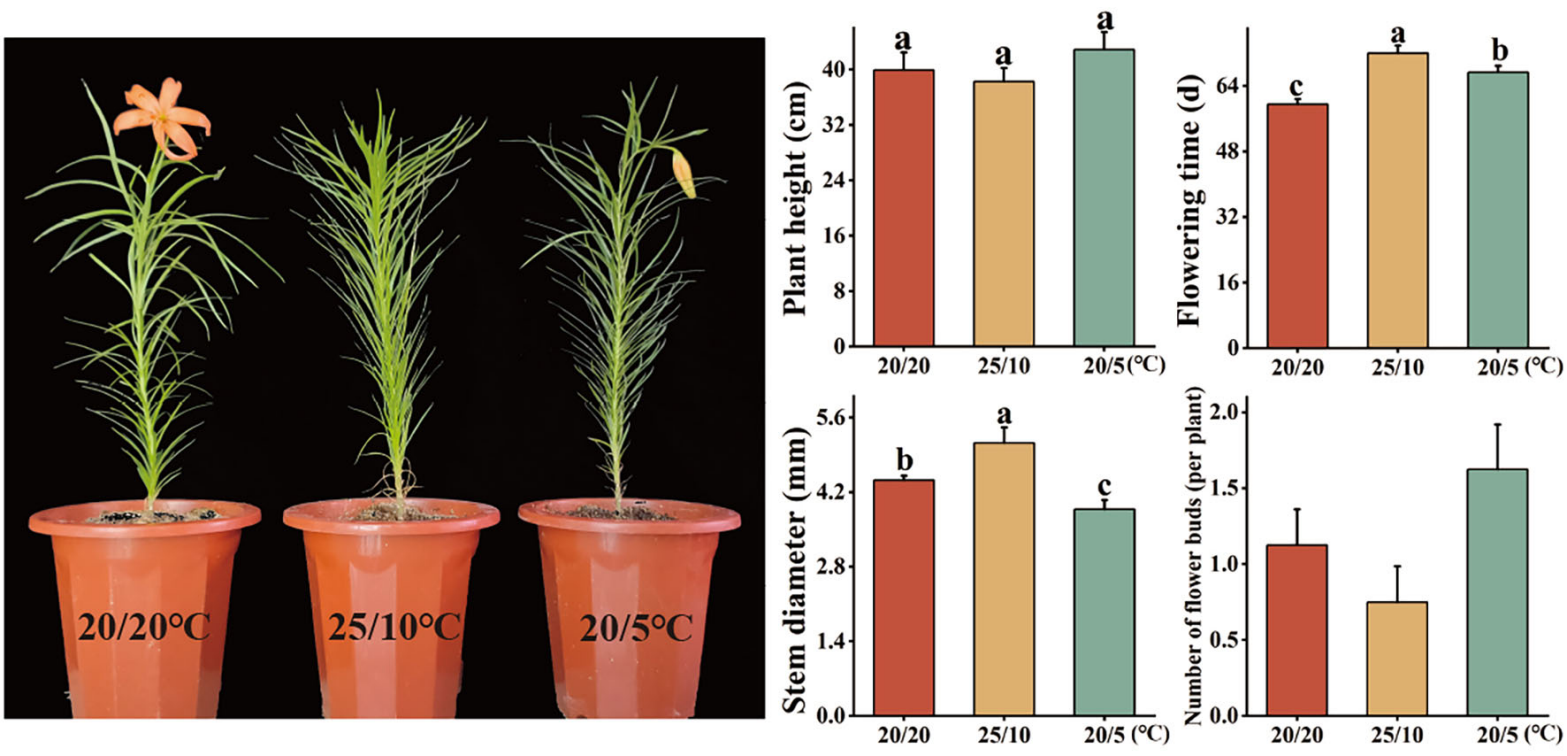
Phenotypic analysis related to the flowering period of the Lanzhou lily. Different lowercase letters in the figure indicate significant differences at the *P* < 0.05 level.

To explore the influence of different diurnal temperatures on the reproductive growth of Lanzhou lily, relevant indicators were measured during the flowering period in this study. The results revealed that during the later stage of plant growth and development, there was no significant difference in plant height among the different diurnal temperature treatments, but the flowering time was significantly delayed. Among them, under the 25°C/10°C and 20°C/5°C treatments, the average flowering period was delayed by 12.5 and 7.8 days, respectively. At 25°C/10°C, the stem diameter increased significantly, by 0.7 mm, whereas at 20°C/5°C, the stem diameter decreased by 0.55 mm. In addition, the total number of flower buds was greatest in the 20°C/5°C treatment, whereas the lowest number was detected in the 25°C/10°C treatment (**Figure 7**). Furthermore, measurements of bulb weight and soluble sugar content, key indicators of bulb nutrient accumulation, provided deeper insights. The 25/10°C treatment resulted in the highest bulb fresh weight (10.45 g) and soluble sugar content (30.09 mg/g), which significantly surpassed those of the control (20/20°C: 8.54 g, 22.47 mg/g). In contrast, compared with the control treatment, the 20/5°C treatment resulted in a bulb weight of 8.97 g, with a sugar content of 24.62 mg/g, indicating a less pronounced effect (**Supplementary Table S2**). These results indicate that although a diurnal temperature difference of 25°C/10°C delays flowering and reduces the number of flower buds, it significantly promotes the thickening of the stems and facilitates the accumulation of biomass and sugars in the bulbs. Therefore, an appropriate combination of diurnal temperature differences might promote the development of Lanzhou lily bulbs.

### 3.8 Investigation of expression patterns in the Lanzhou lily across various conditions

On the basis of published transcriptome data analysis for different tissues of Lanzhou lily, *LdELF4.1* was constitutively highly expressed in all the examined tissues. In contrast, *LdELF4.3* displays marked tissue specificity, with significant upregulation detected exclusively in petals, whereas *LdELF4.4* is highly expressed specifically in anthers (**Supplementary Figure S1**). These findings provide a solid foundation for in-depth investigations into the functional divergence of *LdELF4* gene family members.

**Figure 8:**
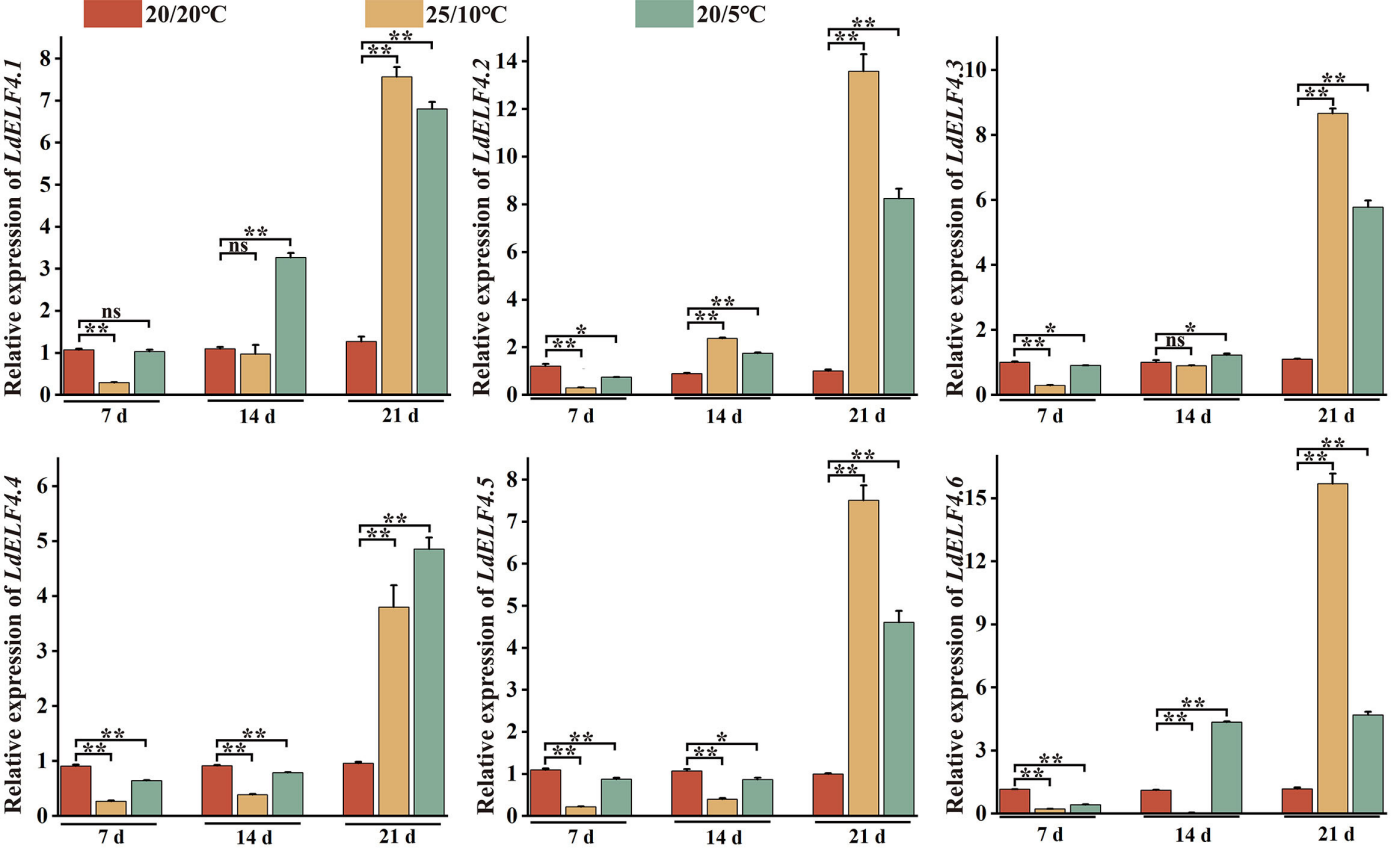
Analysis of *LdELF4* expression patterns under different diurnal temperature treatments. Gene expression levels were determined by qRT–PCR with three biological replicates and normalized to the *LdActin* reference gene. The data are presented as the mean ± SD. Different lowercase letters above the bars indicate statistically significant differences among treatment groups at the same time point, as determined by one-way ANOVA followed by Duncan’s multiple-range test (*P* < 0.05). * indicates *P* < 0.05, and ** indicates *P* < 0.01.

Furthermore, to investigate the response mechanisms of *ELF4* family members to DIFs, the expression dynamics of six *LdELF4*s in *L. davidii* var. *willmottiae* under different diurnal temperature regimens and treatment durations were systematically analyzed using qRT–PCR. The results demonstrated that during the early stages of temperature stress (7 and 14 d), the *LdELF4* expression levels were generally low, suggesting that LdELF4s may not yet be fully activated or may initially maintain basal expression levels. By day 21, however, overall expression increased significantly. Notably, distinct temporal expression patterns emerged among the gene members across the different temperature treatments: *LdELF4.1*, *LdELF4.3*, and *LdELF4.6* presented rapid increases in expression starting on day 21 under the 25/10°C regime. In contrast, under the lower nocturnal temperature treatment (20/5°C), the expression of these genes increased significantly earlier, peaking on day 14, indicating that the expression of these three genes is induced by low-temperature stress. *LdELF4.4* and *LdELF4.5* displayed rapid increases in expression on day 21 under both diurnal temperature treatments, indicating no significant response to the variation in temperature. *LdELF4.2* differed from the aforementioned genes, exhibiting a rapid increase in expression on day 14 under both temperature regimens, similar to the lack of a specific response to DIF (**Figure 8**). Overall, *LdELF4.6* presented the most sensitive expression pattern in response to changes in DIF, suggesting its putative role as a key regulator of Lanzhou lily adaptation to diurnal temperature fluctuations and growth regulation.

## 4 Discussion

### 4.1 Nuclear and cytoplasmic anomalies during the process of species evolution

Plant evolution involves the coordinated evolution of three distinct genetic systems: the nuclear genome, the chloroplast genome, and the mitochondrial genome. To date, research has focused primarily on independent phylogenetic analyses of the chloroplast (Li et al., 2022; Lei et al., 2025; Jiang et al., 2025) and nuclear (Xu et al., 2024; Liang et al., 2025) genomes. On the basis of the assembled genomic data, in this study, the phylogenetic topologies of nuclear and chloroplast genomes across multiple *Lilium* species, garlic (*Allium sativum*), and representative monocot and eudicot species were systematically compared, and the phenomenon of cytoplasmic–nuclear discordance among in these species were investigated. Consistent with widespread reports in angiosperms (Zhang et al., 2023; Hu et al., 2023), substantial discordance between the nuclear and chloroplast genomes was observed. The nuclear genome-based phylogeny conformed to established evolutionary expectations: when *Physcomitoria patens* was used as the outgroup, angiosperms were clearly divided into monocot and eudicot clades (**Figure 1**). Within this framework, the three *Lilium* species and garlic exhibited close affinities, supporting previous conclusions regarding the sister relationship between Liliaceae and Amaryllidaceae (Jin et al., 2020). Pairwise comparison of the nuclear and chloroplast phylogenetic trees revealed a specific subclade comprising three *Lilium* species and garlic that exhibited significant cytoplasmic–nuclear discordance. The phylogenetic placement of this subclade within both the nuclear and plastid trees was distinct from the primary divergence between the monocot and eudicot lineages. This finding might be due to the relatively high conservation of the chloroplast genome, yet its diversity is lower than that of the nuclear genome. Notably, previous phylogenetic studies of *Lilium* chloroplast genomes were largely confined to intrafamilial or intrageneric levels within Liliaceae. This study is the first to reconstruct their phylogeny within a broader evolutionary framework encompassing diverse representative monocot and eudicot plants. Divergence time estimation for this key lineage (the *Lilium*-garlic clade) supports its early history of being characterized by a relatively rapid radiation event. This rapid diversification process was likely accompanied by hybridization events and incomplete lineage sorting (ILS) (Feng et al., 2022); other mechanisms could also contribute to this phenomenon. For instance, chloroplast capture following hybridization could lead to the fixation of a chloroplast genome from one species in the nuclear background of another, creating a discordant phylogenetic signal. Additionally, differential evolutionary rates between the fast-evolving nuclear genome and the more conserved chloroplast genome might result in inconsistent tree topologies. It is also important to consider that methodological artifacts, potentially exacerbated by the current limited sampling of available lily genomes, could influence the resolution of deep nodes. While our data within the existing framework best support a scenario involving hybridization and ILS, future studies with expanded taxonomic sampling will be crucial to fully disentangle the relative contributions of these alternative hypotheses.

### 4.2 Evolutionary relationships of the *ELF4* family

The *ELF4* gene is indispensable for photoperiod perception, circadian clock regulation, temperature response, and rhythm maintenance in plants and has been highly conserved throughout evolution (Doyle et al., 2002; McWatters et al., 2007; Kolmos and Davis, 2007; Kolmos et al., 2009). In this study, members of the *ELF4* family were identified across various species on the basis of their characteristic DUF1313 domain, revealing a fluctuation in membership number, primarily between 3 and 10. This variation may be associated with differences in selection rates or evolutionary pressures among species. Six *LdELF4* family members were identified in Lanzhou lily, a number that is consistent with its diploid evolutionary mode. Phylogenetic analysis separated the *ELF4* family into four distinct subclades (**Figure 2**). Subfamily II predominantly comprises dicot species, potentially reflecting a characteristic inherited from their common ancestor (Fan et al., 2017; Li et al., 2024). In contrast, subfamily III is composed primarily of monocot species, with clustering branches showing both continuity and dispersion, indicating that the ELF4 domain itself is relatively conserved and ancient. The distribution pattern of *ELF4*s across different subclades suggests potential functional divergence, suggesting that genes within the same subfamily may possess similar functions owing to their close evolutionary relationship. Chromosomal localization revealed an uneven distribution of *ELF4* family members across the chromosomes of the three lily species studied (**Figure 3**). Notably, the distribution patterns of Lanzhou lily and *L. sargentiae* were highly similar, providing further evidence for their close evolutionary relatedness. The localization of *G. superba ELF4*s solely to scaffold sequences rather than to assembled chromosomes is likely attributable to the absence of a chromosome-level genome assembly. Whole-genome synteny analysis revealed significant syntenic relationships between Lanzhou lily and *L. sargentiae*, with some chromosomes exhibiting regular correspondence; notably, numerous syntenic blocks were identified between LG08 of Lanzhou lily and Chr5 of *L. sargentiae*. Most *ELF4* family members contain only 2–3 exons (**Figure 4**). The similarity in exon–intron structures among closely related genes aligns with their high homology in the protein phylogenetic tree, suggesting a degree of conservation in these structural elements during evolution (Wang et al., 2021). Furthermore, the acquisition of introns during evolution is a widespread phenomenon, increasing gene structural complexity to accommodate diverse changes (Han et al., 2018). The type and abundance of cis-acting elements within the promoter regions of Lanzhou lily *ELF4*s varied (**Figure 5**). These differences likely underpin their specific roles and functions in distinct physiological processes, such as plant growth and development, hormonal responses, and stress adaptation.

### 4.3 Effects of the diurnal temperature difference on the *LdELF4* members

Members of the *ELF4* family play a role in DIF by regulating plant flowering in response to external light and temperature cues (Jung et al., 2020). As a critical factor influencing flowering in Lanzhou lily, low nocturnal temperatures may affect the expression of *LdELF4*s. Quantitative analysis of published transcriptome data revealed the following tissue-specific expression patterns of *LdELF4*s: *LdELF4.1* was highly expressed across all tissues, *LdELF4.3* was highly expressed in petals, and *LdELF4.4* was highly expressed in anthers (**Supplementary Figure 1**). However, the expression of three genes was undetected, likely because the levels fell below the threshold of detection. This tissue-specific expression pattern aligns with findings in cotton, in which *LdELF4*s were predominantly expressed in reproductive organs (stamens, pistils, and petals) and less so in leaves (Tian et al., 2021). Research has indicated that growth inhibition and cold acclimation strategies help plants withstand cold stress, albeit with detrimental effects on growth and survival (Jiang et al., 2020; Mingzhai et al., 2025). For example, low-temperature stress significantly suppresses the growth and development of winter wheat, resulting in slow seedling growth, reduced plant height, decreased tiller number, and poor root development (Devi et al., 2023). Conversely, studies on maize seedling root tips revealed that compared with 30°C, moderately low temperatures (20°C) promoted greater root elongation and growth (Friero et al., 2023). Furthermore, GA biosynthesis in barley seeds is inhibited under low-temperature stress; however, upon warming to moderately low temperatures, shifts in GA synthesis and metabolism may promote plant growth and development. These findings are consistent with our results, which demonstrated that moderately low temperatures promote *Lilium* growth and development, whereas low-temperature stress inhibits such growth and development (**Figure 7**). Further investigation into the temporal expression patterns of *LdELF4*s under varying diurnal temperatures revealed distinct expression profiles for *LdELF4.1*, *LdELF4.3* and *LdELF4.6*. Under low-temperature stress, these genes presented earlier expression, peaking at 14 days (**Figure 8**). This phenomenon may be attributed to the temperature-dependent mobility of the ELF4 protein. At relatively low temperatures (e.g., 5°C nocturnal temperature), *ELF4* translocation from shoots to roots is relatively efficient, prolonging the root circadian period and leading to ELF4 protein accumulation (Chen et al., 2020). This accumulation potentially triggers compensatory upregulation of shoot *ELF4* gene expression via negative feedback regulation. In contrast, a nocturnal temperature of 10°C is insufficient for full stabilization of the EC but exceeds the optimal temperature threshold. Under these conditions, *ELF3* undergoes partial inactivation, which requires a longer period for *ELF4* accumulation to restore the function of the EC complex (Silva et al., 2020).

## 5 Conclusion

Through systematic nuclear and plasmic genome analysis, this study revealed that there are cytonuclear conflicts in the genus *Lilium* and revealed 62 *ELF4* homologous genes in 11 angiosperms, which were classified into 4 subfamilies. On the basis of the results of the homolinear analysis, the distribution patterns of six *LdELF4*s located at LG01, LG08, LG09 and LG12 in Lanzhou lilies revealed chromosomal structural variations between them and the *L. sargentiae* genome. qRT–PCR analysis indicated that *LdELF4.6* was most sensitive to temperature changes and might respond to diurnal temperature differences. Key phenotypic analysis revealed that, compared with the control treatment at a constant temperature of 20°C, the 25/10°C temperature difference treatment significantly delayed the flowering of Lanzhou lilies by 12.5 days while reducing the number of flower buds and increasing the stem diameter to 5.1 mm. On the basis of the above results, *LdELF4.6* was identified as the key gene that regulates the response of Lanzhou lily to diurnal temperature differences, and this study revealed that 25/10°C is the optimal temperature for bulb development. This study provides important genetic resources and a theoretical basis for the breeding of new varieties of climate-adapted lilies.

## Data Availability

The datasets presented in this study can be found in online repositories. The names of the repository/repositories and accession number(s) can be found in the article/**Supplementary Material**.
