# Supplementary material for: Whole-genome identification of LdELF4s and analysis of their expression response to diurnal temperature variations in Lilium davidii var. willmottiae (E. H. Wilson) raffill
Source: Front Plant Sci. 2026 Jan 7;16:1683733. doi: 10.3389/fpls.2025.1683733 (PMC12819689; doi:10.3389/fpls.2025.1683733)
Supplement: Supplementary file 1 [file DataSheet1.docx]

**Table S1 Sequences of the primer pairs used in the qRT‒PCR analysis**

| **Gene name** | **Primer secquence (5’-3’)** |
| --- | --- |
| *Ld1-F* | ACGAGAACCACCAGTCGAAG |
| *Ld1-R* | TCTTCTTCTTCCCCGGAACC |
| *Ld2-F* | TCGGCCTGATTTACGAGCTG |
| *Ld2-R* | TTCGATGAGGTTGACGAGGC |
| *Ld3-F* | AGAAGAGCTTCGTGCAGGTG |
| *Ld3-R* | CGACCCGCCGGATATTGTT |
| *Ld4-F* | AACATCCTCGACCAGAACCG |
| *Ld4-R* | ACGAAACAGAGAGATCGCCG |
| *Ld5-F* | CGTTCCAGAACAGCTTCGTG |
| *Ld5-R* | CGCCGGATGTTGTTGTTCAG |
| *Ld6-F* | AGATCCCGACCAACTTGAGC |
| *Ld6-R* | GCCTTCTGTGCCTCCAAAAC |
| *LdAct-F* | TGAGCACATTCCAGCAGA |
| *LdAct-R* | CCATAGACAAAGCCATCG |


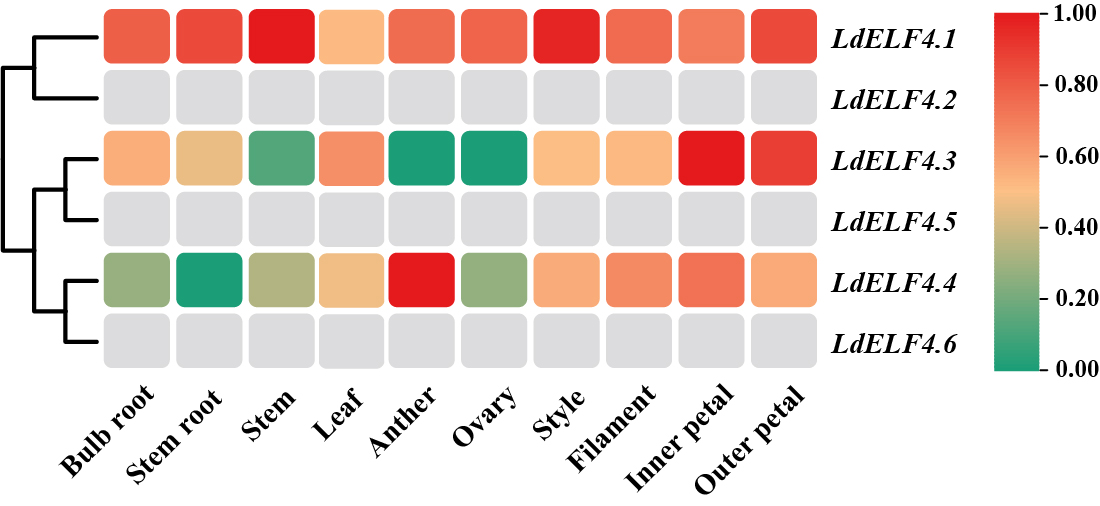
 FIGURE S1

Analysis of the expression pattern of *LdELF4* in different tissues of Lanzhou lily

**Table S2 Bulb weight and soluble sugar content of Lanzhou lily under different diurnal temperature treatments**

| Treatment | bulb fresh weight (g) | soluble sugar content (mg/g) |
| --- | --- | --- |
| 20/5℃ | 8.97±0.32 | 24.62±4.22 |
| 25/10℃ | 10.45±0.83 | 30.09±3.28 |
| 20/20℃ | 8.54±0.89 | 22.47±1.89 |
